# Development of a self-limiting model of methotrexate-induced mucositis reinforces butyrate as a potential therapy

**DOI:** 10.1038/s41598-021-02308-w

**Published:** 2021-11-25

**Authors:** A. R. da Silva Ferreira, S. A. J. van der Aa, T. Wehkamp, H. R. Wardill, J. P. ten Klooster, J. Garssen, L. F. Harthoorn, A. Hartog, H. J. M. Harmsen, W. J. E. Tissing, J. van Bergenhenegouwen

**Affiliations:** 1 Department of Medical Microbiology and Infection prevention, University of Groningen, University Medical Center Groningen, Hanzeplein 1 EB80, 9713 GZ Groningen, The Netherlands; 2 Department of Pediatric Oncology, University of Groningen, Beatrix Children’s Hospital, University Medical Center Groningen, Groningen, The Netherlands; 3 Danone Nutricia Research, Utrecht, The Netherlands; 4 Adelaide Medical School, The University of Adelaide, Adelaide, Australia; 5 Precision Medicine Theme, South Australian Health and Medical Research Institute, Adelaide, Australia; 6 Division of Pharmacology, Faculty of Science, Utrecht Institute for Pharmaceutical Sciences, Utrecht University, Utrecht, The Netherlands; 7 Research Centre for Healthy and Sustainable Living, Innovative Testing in Life Sciences and Chemistry, University of Applied Sciences, Utrecht, The Netherlands; 8 Princess Maxima Center for Pediatric Oncology, Utrecht, The Netherlands

**Keywords:** Chemotherapy, Intestinal stem cells, Microbiome

## Abstract

Gastrointestinal mucositis is a complication of anticancer treatment, with few validated in vitro systems suitable to study the complex mechanisms of mucosal injury. Therefore, we aimed to develop and characterize a chemotherapeutic-induced model of mucositis using 3D intestinal organoids. Organoids derived from mouse ileum were grown for 7 days and incubated with different concentrations of the chemotherapeutic agent methotrexate (MTX). Metabolic activity, citrulline levels and cytokine/chemokine production were measured to determine the optimal dosage and incubation time. The protective effects of folinic acid on the toxicity of MTX were investigated by pre-treating organoids with (0.0005–50 µg/mL) folinic acid. The impact of microbial-derived short-chain fatty acids was evaluated by supplementation with butyrate in the organoid model. MTX caused a dose-dependent reduction in cell metabolic activity and citrulline production that was salvaged by folinic acid treatment. Overall, MTX causes significant organoid damage, which can be reversed upon removal of MTX. The protective effect of folinic acid suggest that the organoids respond in a clinical relevant manner. By using the model for intervention, it was found that prophylactic treatment with butyrate might be a valuable strategy for prophylactic mucositis prevention.

## Introduction

Cancer therapy-induced mucositis is defined as inflammation and consequent damage of the mucosa and submucosal lining of the gastrointestinal tract^1^. While oral mucositis has been studied in greater detail, reflecting the ease at which the oral cavity can be accessed, research that focuses on gastrointestinal mucositis (GI-M) remains limited. Patients undergoing chemotherapy present a spectrum of gastrointestinal symptoms relating to mucositis, including nausea/vomiting, diarrhea, abdominal pain and infection. In their mild form, these symptoms have a significant psychological impact on patients due to fatigue, pain, embarrassment and fear^2–4^. In severe cases, symptoms often require significant supportive care (pain management, parenteral nutrition, anti-microbial agents), compromising quality of life. In extreme cases, chemotherapy dose reductions or complete cessation of treatment are required, compromising patients ‘survival^1^.

Mucositis development is currently proposed to involve five interactive and overlapping phases: (1) the initiation phase, characterized by direct cytotoxicity underpinned by irreversible DNA damage and activation of nuclear factor kappa B (NF-кB), (2) the induction of messenger molecules, resulting in downstream immune signaling and the production of pro-inflammatory cytokines including tumor necrosis factor (TNF) and interleukin 6 (IL-6), (3) amplification of messenger molecules that exacerbate tissue injury, (4) ulceration and breakdown of the epithelial barrier, and (5) spontaneous healing, characterized by cell proliferation^2^. While this pathobiological model provides key foundational knowledge, it fails to acknowledge the complex interactions that govern mucosal injury caused by specific chemotherapeutic agents, limiting our ability to identify translational targets for intervention.

Methotrexate (MTX) is a folate antagonist commonly used, alone or in combination, with other chemotherapeutic drugs in adult and pediatric patients. MTX acts by interfering with the synthesis of folate and thymidine monophosphate, which is a nucleotide (pyrimidine) required for DNA synthesis. As a result, MTX inhibits DNA synthesis thus interfering directly with cell division. This aspect of MTX action is beneficial for the treatment of rapidly dividing malignant cells, however, it is detrimental to the normal functioning of organs that contain cells with a higher turnover, such as epithelial cells lining the intestinal tract^5^. Similarly to other cytotoxic agents, MTX leads to the disruption of the epithelial barrier culminating in an exacerbated inflammatory response^6^.

Currently, clinical and preclinical approaches to model MTX toxicity are limited, with animal models being expensive, time consuming and ethically challenging, and single cell culture models over simplified and failing to recapitulate complex host-microbe interactions that drive mucosal injury. These limitations impact our ability to develop and rapidly screen new interventions for GI-M. In contrast, organoid models are increasingly recognized for their superiority, and are now described for numerous organ systems including the liver, kidney, pancreas, brain and intestine^7–11^. Organoid systems are a powerful tool to study disease processes due to the stem cells ability of self-renewal and to differentiate into specialized intestinal cell types^7, 9, 12^. Despite these clear advantages, currently there is no organoid models for the study of MTX-induced mucosal injury. As such, we aimed to develop and characterize an organoid model of chemotherapy induced GI-M and assess the efficacy of microbial metabolites.

## Results

### 3D-intestinal mouse organoids maintain the characteristics of the tissue of origin

The organoid model was developed based on methods described by Sato et al., 2009^11^. Organoids cultured in this fashion were also characterized to harbor different epithelial cell types^13^. These organoid structures contain all intestinal cell types such as stem cells, paneth, goblet, endocrine cells and to a lower extend also enterocytes. The latter are mainly present when organoids are cultured in 2D on collagen I-coated plates^13^. However, in this study the BMP4 inhibitor Noggin was replaced by DMH1, a pharmaceutical which also inhibits BMP4. To confirm that cultures contain all cell types, the expression of cell-specific markers in 3D and 2D grown organoid cultures was analyzed (Figure S1). In agreement with previously described data, expression of Stem, Paneth, Goblet and endocrine cell-specific markers in 3D was found, which was reduced in 2D. Markers for enterocytes, although present in 3D, were increased when organoids were cultured in 2D (Figure S1). This indicates that organoid cultures contain adult stem cells which can still differentiate into all specific intestinal cell lineages.

### MTX affects metabolic activity and citrulline production in a dose-dependent manner

**Figure 1:**
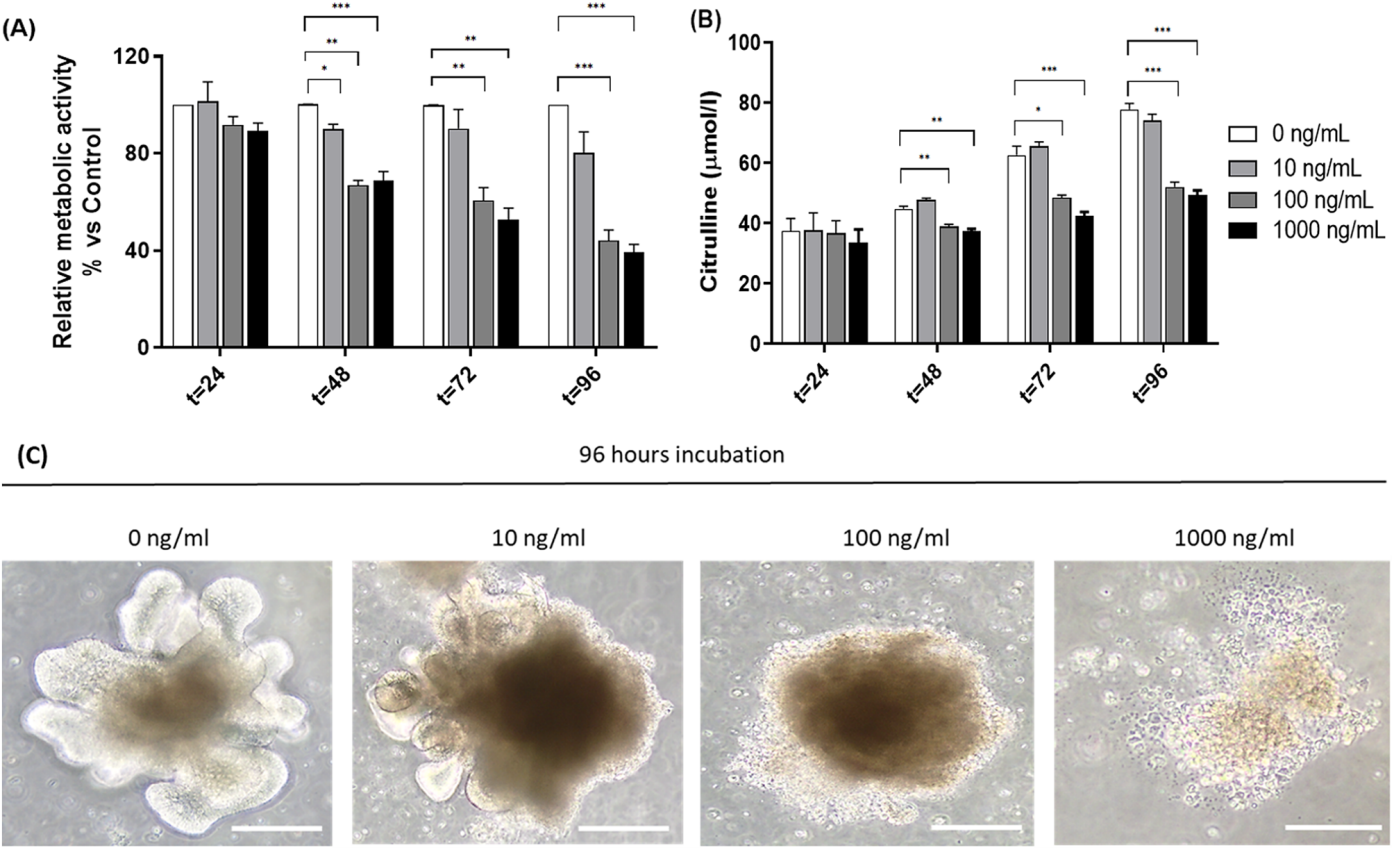
Evaluation of methotrexate-induced cytotoxicity in organoids. Organoids were treated with different concentrations of MTX (0, 10, 100 and 1000 ng/mL) for different time points (24, 48, 72 and 96 h). (**A**) Relative metabolic activity of organoids treated with or without MTX (n = 6). (**B**) Citrulline levels in culture supernatants collected at 24, 48, 72 and 96 h of incubation with MTX (µmol/l) (n = 4). (**C**) Optical microscopy of MTX-treated organoids for 96 h (40x). Scale bar = 200 μm. All data are presented as mean ± SEM. *(*p* < 0.05), **(*p* < 0.01) and ***(*p* < 0.001) represent the significant changes between MTX-treated and untreated organoids.

MTX induced dose-dependent mucosal injury in the organoids (Fig. 1). Incubation with 10 ng/mL MTX at different time points had no effect on metabolic activity relative to untreated organoids, as indicated by WST-1. In contrast, treatment with 100 ng/mL and 1000 ng/mL MTX resulted in a significant reduction in metabolic activity after 96 h of incubation, with a relative metabolic activity of 44% and 39%, respectively (P = 0.0007 and P = 0.0002; Fig. 1A). An increase in the concentration of citrulline in untreated organoids was observed demonstrating continuing expansion of enterocytes in the organoids (Fig. 1B). In contrast to 10 ng/ml, 100 and 1000 ng/ml MTX induced a significant reduction in citrulline levels (P = 0.0002 and P = 0.0001, respectively; Fig. 1B). This was accompanied by clear morphological damage as indicated by loss of 3D structure (Fig. 1C).

### MTX-treated organoids fully recover after refreshment of media

**Figure 2:**
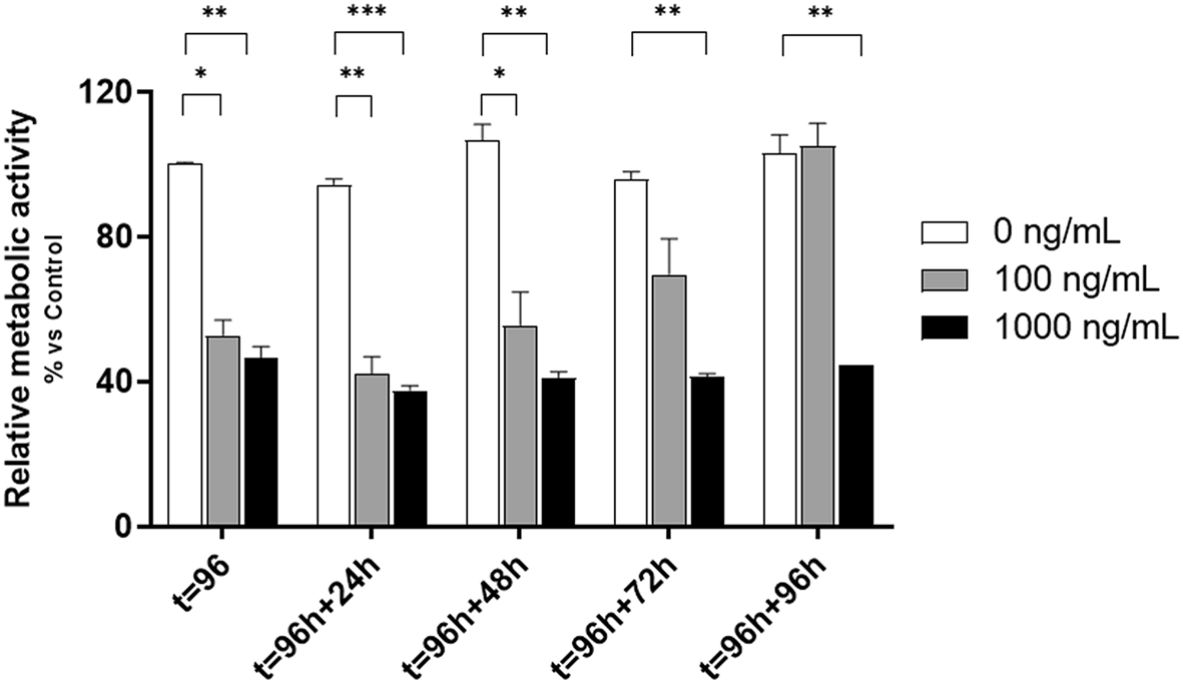
Relative metabolic activity after media refreshment. After 96 h of incubation, medium containing MTX was washed away and fresh media was added. Supernatant was collected at 24, 48, 72 and 96 h and WST-1 performed (n = 3). All data are presented as mean ± SEM. * (*p* < 0.05), ** (*p* < 0.01) and *** (*p* < 0.001) represent the significant changes between MTX-treated and untreated groups.

Clinically, GI-M is a self-limiting phenomenon, with mucosal recovery occurring after completion of cytotoxic therapy. To confirm this attribute in our model, the media was refreshed after 24–96 h and organoids were left to recover. Their activity was determined by WST-1. Organoids treated with 1000 ng/mL MTX were unable to recover after 96 h of incubation with fresh medium (P = 0.009; Fig. 2). In contrast, organoids treated with 100 ng/mL MTX showed significant recovery over time, as reflected by an increase in metabolic activity (Fig. 2). Based on this we choose 100 ng/ml for next experiments, as the effect of this concentration resembled the clinical situation the most.

### Folinic acid reverts MTX effects in a dose and time-dependent manner

**Figure 3:**
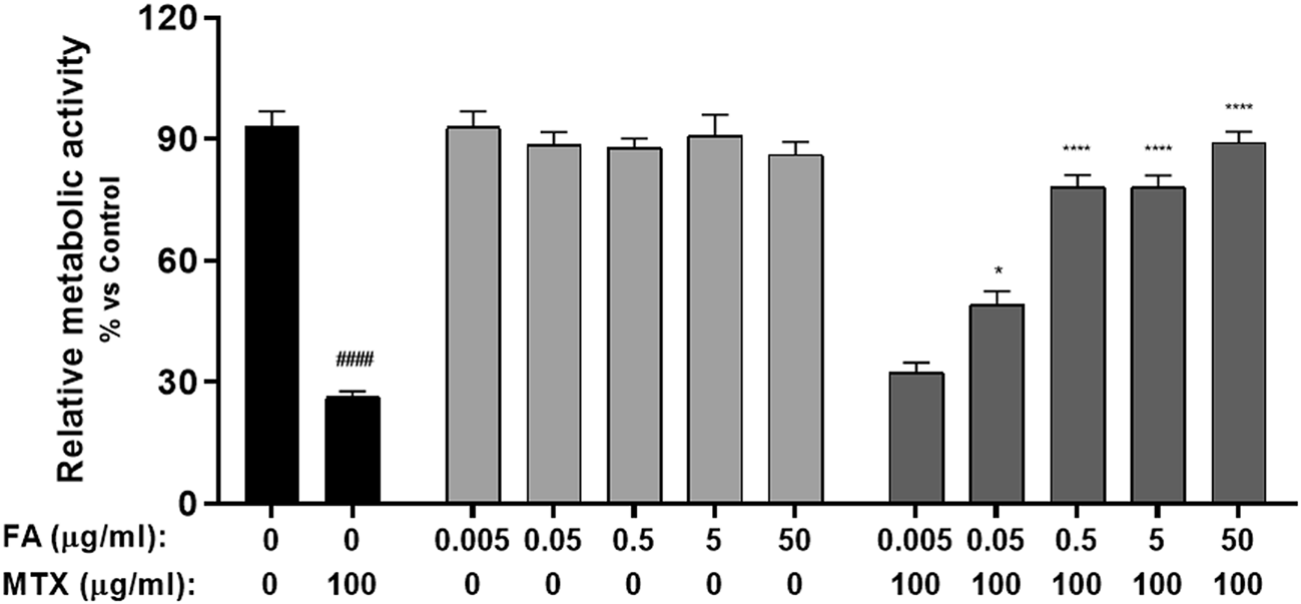
Folic acid dose response in MTX-induced mucositis organoids. Relative metabolic activity of organoids treated with 100 ng/mL MTX and the simultaneous addition of 0.005, 0.05, 0.5, 5 and 50 µg/mL folinic acid for 96 h (n = 3). Data is presented as mean ± SEM. #*p* < 0.05 for control organoids versus MTX-treated organoids. *p < 0.05 and *p* < 0.0001 for MTX-treated organoids versus MTX and FA-treated organoids.

Firstly, the optimal concentration of folinic acid (0.005-50 μg/ml) to rescue MTX-treated organoids was determined. After 96 h of incubation with 100 ng/mL MTX, organoids showed a significant decrease in the relative metabolic activity, as previously observed (*p* < 0.0001; Fig. 3). In organoids treated only with folinic acid, no significant differences in metabolic activity were observed at any of the concentrations tested (Fig. 3). When organoids were simultaneously treated with both MTX and folinic acid, a significant inhibition in the decrease of metabolic activity was observed at doses 0.05, 0.5, 5 and 50 µg/mL (P = 0.012, *p* < 0.0001, *p* < 0.0001, *p* < 0.0001, respectively; Fig. 3). A folinic acid concentration of 5 µg/mL was selected for the following recovery experiments.

**Figure 4:**
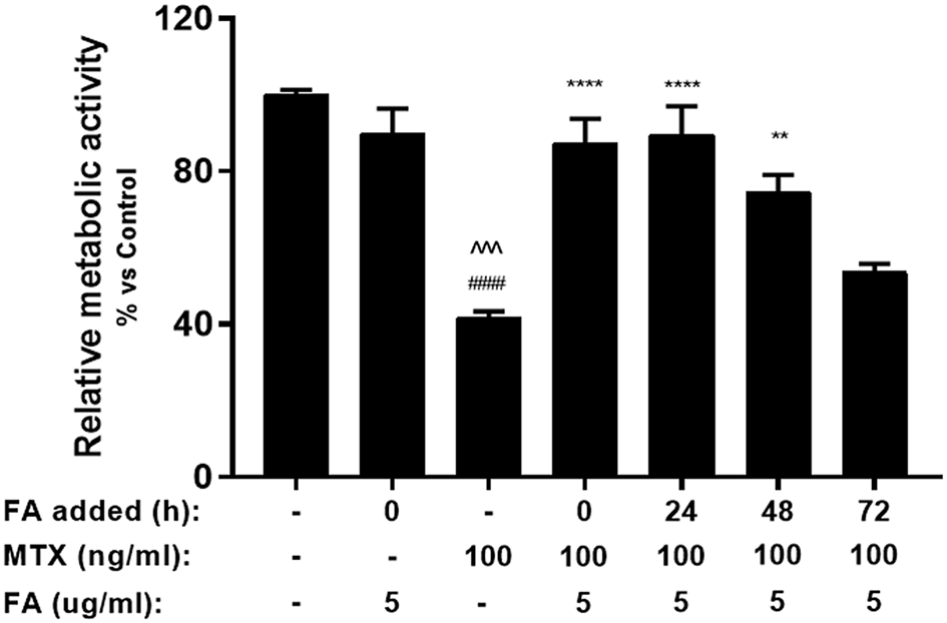
Relative metabolic activity of MTX and FA-treated organoids at different stages of treatment. MTX-treated organoids were supplemented with FA at different time points (0, 24, 48 and 72 h) with 5 µg/mL FA (n = 4). Metabolic activity was measured by WST-1 assay. All data are presented as Mean ± SEM. ####*p* < 0.0001 represents the significant differences observed between untreated organoids and MTX-treated organoids. ^^^*p* < 0.001 represents the significant differences between FA-treated organoids and MTX-treated organoids. **p < 0.01 and ****p < 0.0001 represent the differences observed between MTX-treated organoids and MTX/FA-treated organoids.

Next, we determined the timeframe at which addition of folinic acid was still effective. As previously observed, MTX treatment significantly reduced relative metabolic activity in organoids when compared to the control (*p* < 0.0001). When 5 μg/ml folinic acid was added either simultaneously or 24 h after treatment, a decrease of metabolic activity was prevented (*p* < 0.0001). Comparable results were observed after 48 h of incubation (P = 0.002). When added in a later phase, folinic acid was unable to prevent the damage caused by chemotherapy (Fig. 4).

### MTX induces an inflammatory response in ileal organoids

**Figure 5:**
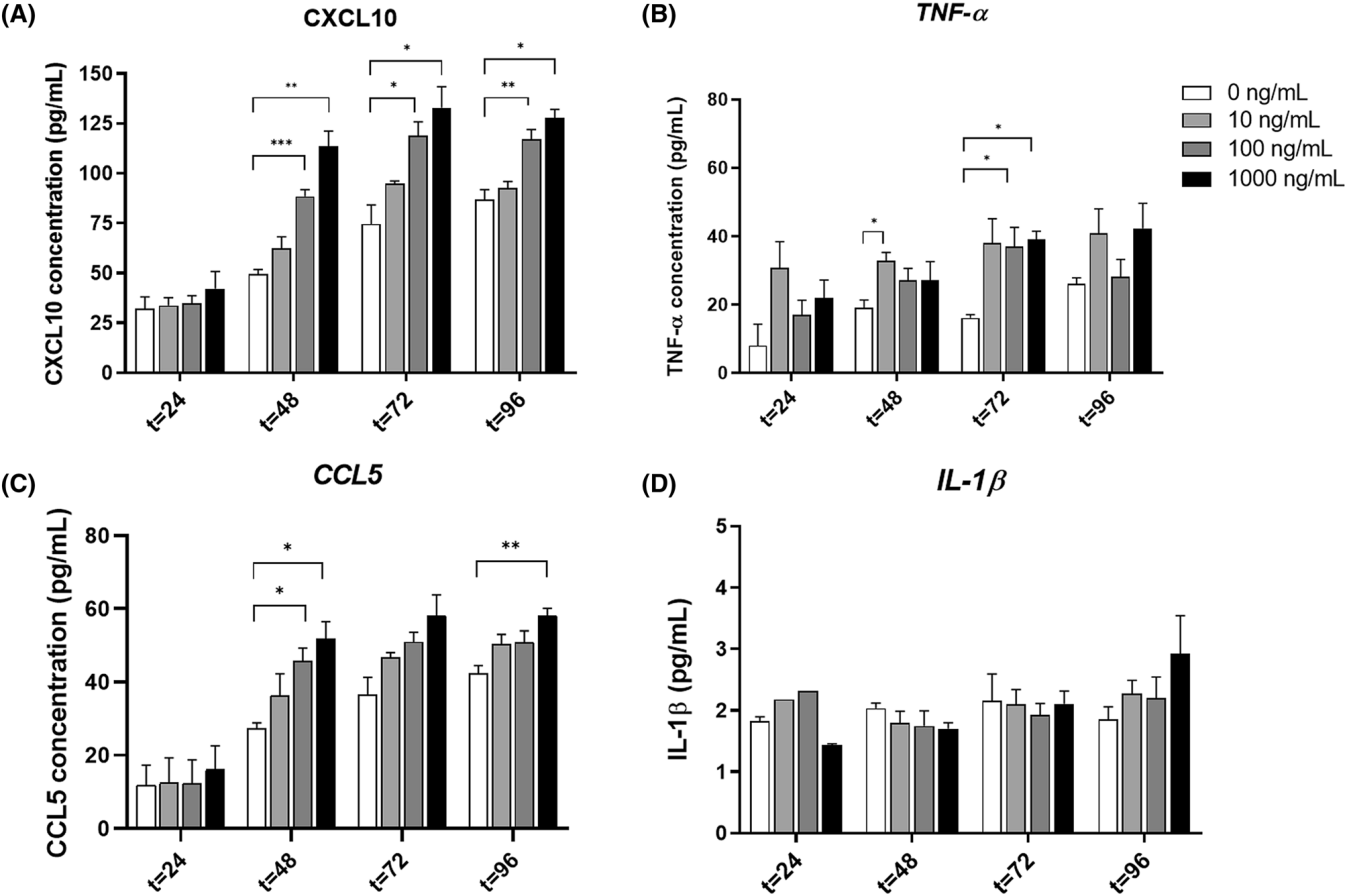
Cytokine and chemokine production in the MTX-induced mucositis organoids. Organoids were treated with 10, 100 and 1000 ng/mL MTX and incubated for 96 h. At different time points supernatant was collected and CXCL10 (**A**), CCL5 (**B**), TNF-α (**C**) and IL-1β (**D**) measured by Multiplex Immunoassay (n = 4). All data are presented as mean ± SEM. * (*p* < 0.05), ** (*p* < 0.01) and *** (*p* < 0.001) represent the significant changes between the MTX-treated and untreated groups.

MTX induced significant changes in CXCL-10, TNFα and CCL5 (Fig. 5). CXCL-10 concentration was significantly increased in the 48-, 72- and 96-h cultures receiving 100 ng/ml (P = 0.0005, P = 0.0434, P = 0.0174, respectively; Fig. 5A). Comparable results were observed with 1000 ng/ml of MTX (P = 0.0070, P = 0.0253, P = 0.0036, respectively). In contrast, a significant increase in TNFα was only observed after 72 h of incubation with 10 ng/ml and 1000 ng/ml MTX (P = 0.026; P = 0.016; Fig. 5B). Chemokine CCL5 was significantly increased in the 48- and 96-h cultures receiving 100 and 1000 ng/mL MTX (P = 0.022; P = 0.021; Fig. 5C). After 96 h of incubation, this increase was only observed at the MTX concentration of 1000 ng/mL (P = 0.0038; Fig. 5C). Remarkably, none of the MTX dosages were able to induce an increase inIL-1β levels at any time point during incubation (Fig. 5D).

### At low doses, butyrate and propionate protect metabolic activity in MTX-treated organoids

**Figure 6:**
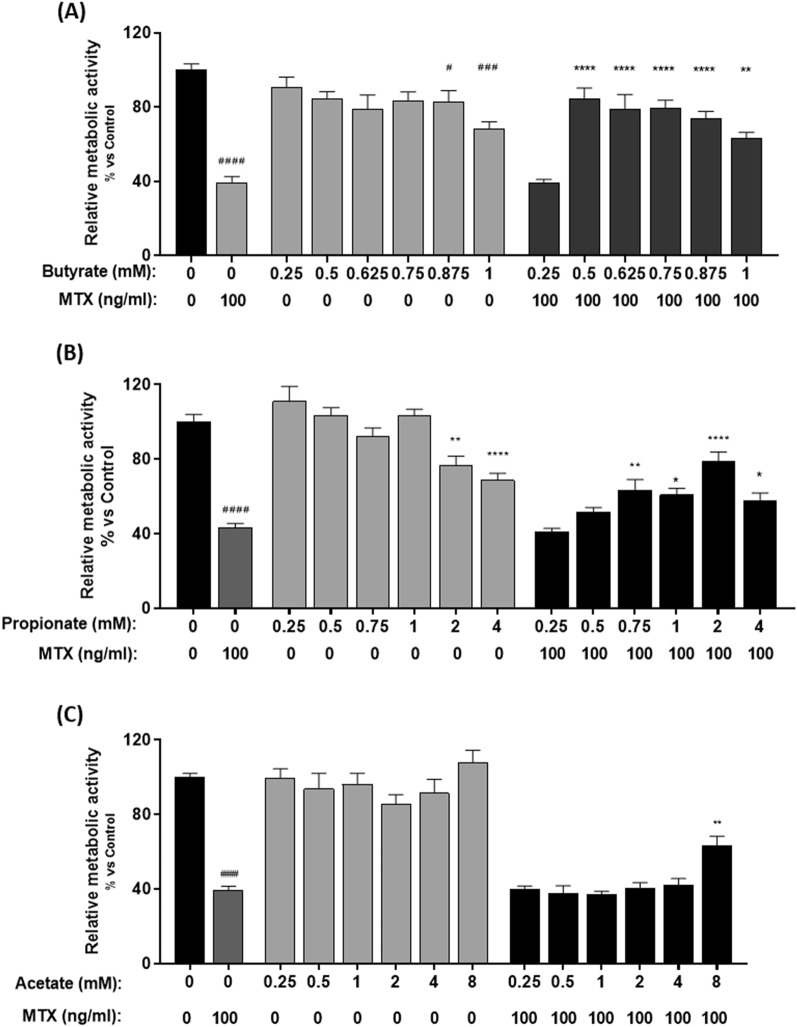
Relative metabolic activity of MTX-treated organoids after butyrate, propionate and acetate administration. Organoids were treated with MTX and different doses of (**A**) butyrate (0.25–1 mM), (**B**) propionate (0.25-4 mM) and (**C**) Acetate (0.25-8 mM) for 96 h. Control groups were treated with different doses of SCFAs (n = 4–7). Relative metabolic activity was measured by WST-1. All data are presented as Mean ± SEM. ####*p* < 0.0001 represents the significant differences observed between untreated organoids and MTX-treated organoids. *p < 0.05, **p < 0.01 and ***p < 0.001 and ****p < 0.0001 represent the differences observed between MTX-treated organoids and MTX/SCFAs-treated organoids.

To test the ability of butyrate, propionate and acetate to prevent organoid damage in this model, organoids were treated with and without MTX (100 ng/ml) and simultaneously supplemented with these SCFAs at different dosages (Fig. 6). The most significant results were obtained with butyrate (Fig. 6A). When organoids were treated with both MTX and butyrate, the MTX-induced decrease in metabolic activity was improved compared to controls treated only with MTX (*p* < 0.0001; Fig. 6A). In the absence of MTX, higher concentrations of butyrate had a negative impact on the metabolic activity (P = 0.001). Similarly, propionate (0.75 to 4 mM) was able to prevent the MTX-induced decrease in metabolic activity, with optimal results observed with 2 mM propionate (*p* < 0.0001; Fig. 6B). Contrary to butyrate and propionate, acetate did not show any significant effects at low doses, with only 8 mM acetate able to prevent MTX-induced injury (P = 0.008; Fig. 6C).

## Discussion

In this study, we report the first organoid model of MTX-induced mucositis, characterized by self-limiting mucosal injury which can be salvaged by the common clinical intervention, folinic acid, as well as by the microbial metabolites butyrate, propionate and acetate.

In line with clinical observations, we showed that MTX affects organoid viability in a dose-dependent manner, mainly characterized by decrease in citrulline production. Moreover, we observed that supplementation of the medium with high levels of folinic acid (5 μg/ml) resulted in the rescue of MTX-treated organoids. Folinic acid supplementation is routine clinical practice, administered 36 h after MTX to quench its mechanism of action. This approach has been shown to enable MTX to have sufficient time to induce its cytotoxic effects, but limits its widespread toxicity. The ability of folinic acid supplementation reiterates the strong parallel between clinical practice and our model and confirms the changes in our model are caused by MTX^14, 15^. Driehuis et al., 2020 very elegantly demonstrated that in an oral model of MTX-induced mucositis, 24-h pretreatment or an earlier start and long continuation with folinic acid (Leucovorin) can reduce MTX-induced cell death^16^. Different from our study, Driehuis et al., 2020 studied oral mucositis using MTX-challenged wild-type human oral mucosa organoids while we have studied MTX-challenged intestinal organoids derived from wild-type mice. However, in both models timely treatment with folinic acid could revert MTX-induced organoid damage which is in-line with clinical observations.

In addition to its clinical relevance, our model has also provided mechanistic insight useful for the development of new interventions. Firstly, we described the inflammatory response upon MTX insult. For that, we measured the levels of a wide range of cytokines/chemokines at different timepoints. To our surprise, only observed significant differences in the levels of TNF-α, CXCL-10 and CCL5, which might be explained by the lack of immune cells in this model. The lack of an immune system could also explain the low expression of IL-1β, as epithelial cells are likely a small source of intestinal inflammation^17–19^. Co-cultures of immune cells with organoids have been described and could extend these observations with investigations into their relative contributions^20, 21^. However, while this is desirable, it is logistically challenging and is beyond the scope of this study.

Based on the current pathobiological understanding of GI-M, both epithelial and immune cells are critical in driving innate immune responses that underpin mucosal injury^22^. As such, the cytokine dynamics of our model reflect only epithelial cytokine products^23, 24^. However, this may also be viewed as an advantage, with dissection of epithelial versus immune cell responses critical in our ability to protect the mucosa without impeding immunogenic cell death, an increasingly recognized aspect of anti-cancer efficacy. This has been shown in the context of TLR4, where epithelial and immune TLR4 are proposed to have differing roles in the context of chemotherapy toxicity and efficacy^25^. By using models where only epithelial processes are occurring, we can begin to identify strategies to prevent mucosal injury without impacting systemic immune tone. Nonetheless, prior to translating this knowledge, these approaches must be evaluated in the context of a co-culture system where immune cells are present. Continued development of our model to include immune cells and other elements of the gastrointestinal microenvironment (e.g., stomal cells and microbes) presents as a key step in generating a pipeline capable of identifying and targeting novel mechanisms.

The concentrations of the chemokines CXCL10 and CCL5 increase in a dose-dependent manner. CXCL10 is mediated by pro-inflammatory stimuli such as microbial products, TNF-α and IFN-γ, and it is involved in the regulation of leucocytes and T-cells, therefore contributing to inflammation^26, 27^. Similar to this chemokine, CCL5 has been shown to promote the recruitment of inflammatory cells, such monocytes, and it has been linked to inflammatory bowel disease^28, 29^. The increased concentration of these chemokines could suggest that specific mucosal inflammatory pathways are being activated leading to increased amounts of TNFα and consequently increased damage of mucosal cells. Interestingly, we also observed a gradual increase in the levels of CXCL10, TNF-α and CCL5 in untreated organoids. A possible explanation for these findings could be that these cytokines accumulate over time in the medium, but it could also represent increased production by more differentiated cells. Higher expression of these chemokines may also lead to reduction of tight junction proteins, which should be considered in future experiments.

While inflammation is a key initiating factor in GI-M, the resident microbiota have also been reported to be critical in governing GI-M by influencing the immune tone, barrier function and drug metabolism^30–33^. The main fermentation products of gut microbial metabolism are short-chain fatty acids (SCFA). Examples of these products are butyrate, propionate and acetate^34^. Butyrate has received greater attention due to its role in regulating the balance between epithelial cell proliferation, differentiation and apoptosis. On the other hand, while propionate is used as a substrate for fatty acid synthesis, acetate was shown to be one of the main products of bifidobacteria, therefore resulting in protection from enteropathogenic infections^35^. In our model we have shown that all three of these SCFAs exert beneficial effects on MTX-treated organoids, although the most profound results were observed for butyrate. These findings are in line with previous studies demonstrating the efficacy of butyrate on inflammatory bowel diseases and on GI-M. In particular, administration of butyrate in mice treated with the chemotherapeutic drug 5-fluorouracil resulted in reduced inflammation and improved cell integrity^36, 37^. Despite the beneficial effects, we also observed that higher concentrations of butyrate and propionate significantly reduce organoid viability. These are in accordance with previous studies that demonstrate that, contrarily to lower concentrations, higher amounts of these SCFAs could lead to cell toxicity. In fact, in a study by Barcelo et al*.* 2000, 5 mM butyrate induced mucus secretion in colonic cells, yet 100 mM butyrate resulted in the inhibition of colonic secretions^38^. This shows that, at specific concentrations, these SCFAs could either prevent or enhance MTX-induced damage.

MTX is an anti-folate drug that penetrates the gastrointestinal tract through carrier-mediated transport system^39^. This drug is absorbed through proton-coupled folate transporters SLC46A1 and SLC19A1 and it effluxes from the cells by members of the ATP binding cassette transporters (ABC transporters), particularly ABCC1, ABCC2, ABCC3, ABCB10, ABCG5 and ABCG8^40, 41^. Previous studies have shown that butyrate is able to bind to ABC transporters, thus conferring protection against colonic inflammation and tumor cell resistance^42^. Based on these findings, we hypothesize that MTX and butyrate have a pharmacodynamic interaction in epithelial cells present in the intestinal organoids. We believe that butyrate binds to ABC transporters in epithelial cells, allowing their overexpression and consequently increase of MTX’s efflux. This results in reduced concentrations of MTX inside the cells and consequently to the prevention of MTX-induced decrease in metabolic activity. Our results could also to some extent explain the observations that severe stages of mucositis seem to be associated with the loss of butyrate-producing bacteria^43, 44^. Indeed, our data could suggest that loss of butyrate would sensitize epithelial cells for MTX leading to increased toxicity and occurrence of mucositis. However, scientific data supporting this theory is currently lacking.

Despite displaying unparalleled similarities with the clinical dynamics of MTX-induced mucositis, our model presents with some limitations that were not explored in the present study. Firstly, our investigations do not show which epithelial cell types are specifically affected by MTX treatment. It would be of interest to research potential separate effects on organoid stem cells versus more differentiated cells. However, we hypothesize that, since MTX acts by preventing the growth of cells with a rapid turnover, it most likely attacks stem cells, therefore explaining the significant reduction in cell viability observed in MTX-treated organoids, when compared to untreated organoids^5, 45^. Secondly, as human starting material was not available to us, we used mouse material to derive organoids from. Future studies should compare human data with mouse data to ensure the translational value of the current findings. Nevertheless, as animal models are still used in mucositis studies, our data are still of value to select potential beneficial pharmaceutical or nutritional components with MTX-protective effects. Thirdly, an important limitation to address is the lack of an immune system. Given that inflammation is a key component of 5-stage process of intestinal mucositis, the addition of immune cells would be of great value to this system.

Overall, our study provides crucial information on mechanisms underlying chemotherapy-induced mucositis. In line with the growing evidence of the protective role of the microbiome in GI-M, we also identify butyrate as a therapeutic strategy. This model therefore offers a simple and robust method to rapidly screen new interventions and to dissect host-microbe interactions during mucositis development.

## Materials and methods

### Culture medium

Organoids were maintained and passaged in WXR medium consisting of Dulbecco’s Modified Eagle Medium F-12 Nutrient Mixture (DMEM/F12) + GlutaMAX (Gibco) with 10% heat inactivated Fetal Bovine Serum (Gibco), 1% Penicillin Streptomycin (Gibco), 1% HEPES 1 M (Invitrogen), 5% Rspondin1 (Homebrew) and 5% WNT (Homebrew) and 0.5 µg/ml DMH1 (Sigma) as a bmp inhibitor^13, 46, 47^. For the first passage of the newly established organoid line, organoids were cultured in VACY medium consisting of WXR medium supplemented with 4.3 mM CHIR99021(Sigma), 10 mM Rho-associated kinase (ROCK) inhibitor Y27632 (Selleckchem), 1 mM 2-Propylpentanoic acid (Sigma)^11, 48^. WXR and VACY medium was kindly provided by the Research Centre for Healthy and Sustainable Living, Innovative Testing in Life Sciences and Chemistry, University of Applied Sciences, Utrecht, The Netherlands.

### Crypt isolation

All methods were carried out in accordance with relevant guidelines and regulations. Experiments were approved by the Animal Experiments Committee of the Utrecht University (AVD108002016394). Crypt isolation was performed according to methods described before^11, 13^. A female mouse C57/BL6, 6 months of age, was sacrificed using carbon dioxide. The intestine was removed and the ileum isolated. Villi were removed from the ileum by scraping and the tissue cut into small pieces, transferred to a tube with ice-cold PBS and washed three times with PBS. Tissue fragmentation was performed using a Polter-Elvehjem tube and the cell suspension filtered through a 100 μm filter. The isolated crypts were spun down at 335 × g for 5 min at room temperature and supernatant removed. The crypts were resuspended in Matrigel (Basement membrane Matrix Growth Factor Reduced Low concentration, Phenol-red-free #356,231, Corning). Drops of 20 μl suspension were added to each well (Corning Costar) and 1 ml of VACY medium added to the wells. Crypts were allowed to differentiate for 3 days at 37˚C and 5% CO_2_. Medium was then replaced by WXR culture medium and cultures allowed to incubate for 4 days in the same conditions.

### Intestinal organoid culture

Matrigel droplets containing isolated crypts were cultured in 24 wells culture plate (Corning Costar) in 1.5 ml of WXR medium. Organoids were grown for 7 days at 37˚C and 5% CO_2_ and their morphology and size were checked every other day. For passaging, organoids containing at least one crypt were counted (40 × magnification) collected by disrupting and scraping the Matrigel droplets followed by shearing through pipetting up and down. The suspension of organoids was visually checked for particle size under the microscope at 400 × magnification. Next, the suspension was centrifuged at 335 × g for 5 min at room temperature, supernatant removed and fresh Matrigel was used to gently resuspend the pellet (80 µl per harvested well). 20 µl of organoid suspension was added to a new pre-warmed (37 °C) 24-wells plate, returned to the CO_2_ incubator for 10 min to settle before the addition of 1.5 fresh WXR medium. Organoids were passaged every 7 days at a 1:4 split ratio. Organoids cultured in this fashion were previously characterized to harbour different epithelial cell types^13^. For biological replicates, experiments were performed with organoids from different passages (N = 3). For a single experiment, in each experimental group, 3 wells containing approximately 40 organoids were included (n = 3).

### Development of the mucositis model

#### MTX dose finding

To select an optimal MTX dose and incubation time, 7 days post-seeding organoids were used. Prior to MTX insult, organoids were counted and only droplets containing 50–80 organoids per well were included in the experiments. Medium was then removed and replaced by fresh WXR medium (900µL). MTX was diluted in new medium until the appropriate concentration. 100µL of the resultant dilutions were added to the organoids with an end concentration of 0, 10, 100 and 1000 ng/ml. Organoids were incubated for different time periods (24, 48, 72 and 96 h) at 37˚C and 5% CO_2_. After incubation, 200µL of medium was removed and stored at -20˚C for citrulline and cytokine/chemokine measurements. Metabolic activity (measured as activity of mitochondrial dehydrogenases) was assessed with WST-1 staining. WST-1 outcomes were considered as cumulative readouts of cell death, loss of cell proliferation and/or metabolic activity, all indicators of organoid loss of function.

#### Recovery assay

To confirm for the similarity to clinical situations, in which MTX causes reversible damage to normal, no cancer cells, we used a recovery assay, For this, MTX-treated organoids to recover from MTX insult, 7-days seeded organoids were first treated with 0, 100 and 1000 ng/ml MTX for 96 h. MTX-containing medium was then removed and the same amount of fresh MTX-free medium was added. Organoid cultures were incubated for 24, 48, 72 and 96 h at 37˚C and 5% CO_2_. At the correspondent time points, 200µL of medium was collected and stored for cytokine/chemokine analysis and metabolic activity was assessed with WST-1.

#### Folinic acid supplementation

To check for the similarity to clinical situations, in which the MTX effect is blocked by adding folinic acid, we studied its effect in our organoid model. To optimize the dose of Folinic Acid (FA) to be used in the experiment, different concentrations ranging from 0.005 and 50 µg/ml were added in combination with MTX (100 ng/ml). Following optimization, organoids were treated with MTX (100 ng/ml) for 96 h and FA (5 µg/ml) was added simultaneously (0 h) or at different time points (24, 48, 72 and 96 h). As previously described, 200µL was removed from the cultures and stored and WST-1 was performed in order to assess metabolic activity.

### Citrulline levels

Citrulline is a validated biomarker of mucosal injury used to quantify GI-M preclinically and clinically. To evaluate the dynamics of this biomarker in our model, culture supernatants were collected and stored at -20˚C degrees until the assay was performed. Proteins and polypeptides were precipitated with perchloric acid and the culture medium was centrifuged. Amino acids, including citrulline, were determined in the supernatant by fully automated High-Performance Liquid Chromatography (HPLC) as previously described^49^.

### Metabolic activity

WST reagent (Cell Proliferation Reagent, Roche Life Science) was added to the medium (1:10) and incubated at 37 °C. At time points 0, 3 and 5 h 100 μl were collected and absorbance was measured at 450 nm and background at 650 nm using Flexstation3 Multi-mode Microplate Reader (Molecular Devices). Absorbance of the samples was corrected for the background.

### Cytokine/chemokine release

Stored culture supernatants were used in this assay. A wide range of cytokines/chemokines were tested in the medium of the organoids including Eotaxin, GM-CSF, Gro-olpha/KC/CINC1, TNF-α, IL-10, IL12p70, IL-13, IL-17A, IL-18, IL-1beta, IL-2, IL-22, IL-23, IL-27, IL-4, IL-5, IL-6, IL-9, MCP-1, MCP-3, MIP1-α, MIP1-β, MIP-2, RANTES, CXCL10 (IP-10) and CCL5. The Multiplex Immunoassay was performed according to the instructions of the manufacturer (Cytokine/Chemokine Panel 1 (26plex), #EPX260-26,088–901, eBioscience). Analysis of the samples was performed using Flexmap3D (Luminex). Recovery of the standards between 70–130% was accepted, values beyond these percentages were considered as invalid.

### Short-chain fatty acids administration

Butyrate, propionate and acetate as used in this study were obtained commercially (Sigma). To assess the ability of different SCFAs to improve relative metabolic activity of MTX-treated organoids, 7-days seeded organoids were treated simultaneously with MTX (100 ng/ml) and butyrate (0.25–2 mM), acetate (0.25-8 mM) or propionate (0.25-4 mM) for 96 h. Medium (200µL) was removed from the wells and WST-1 assay was performed on the remaining media in order to assess metabolic activity.

### Statistical analysis

Data were compared using Prism version 8.0.1 (GraphPad Software, San Diego, USA). Significant differences between different groups were determined by repeated measured Two-Way ANOVA with Turkey’s multiple comparison test or one-way ANOVA with Dunnett’s multiple comparisons test. A p-value < 0.05 was considered statistically significant.

### Ethics approval and consent to participate

This study was performed in compliance with the ARRIVE guidelines. Experiments were approved by the Animal Experiments Committee of the Utrecht University (AVD108002016394).

## Supplementary Information

Supplementary Figure S1.

Supplementary Information.
