# Supplementary material for: Development of a self-limiting model of methotrexate-induced mucositis reinforces butyrate as a potential therapy
Source: Sci Rep. 2021 Nov 25;11:22911. doi: 10.1038/s41598-021-02308-w (PMC8617074; doi:10.1038/s41598-021-02308-w)
Supplement: Supplementary file 1 — Supplementary Figure S1. [file 41598_2021_2308_MOESM1_ESM.docx]

**Supplementary figures**


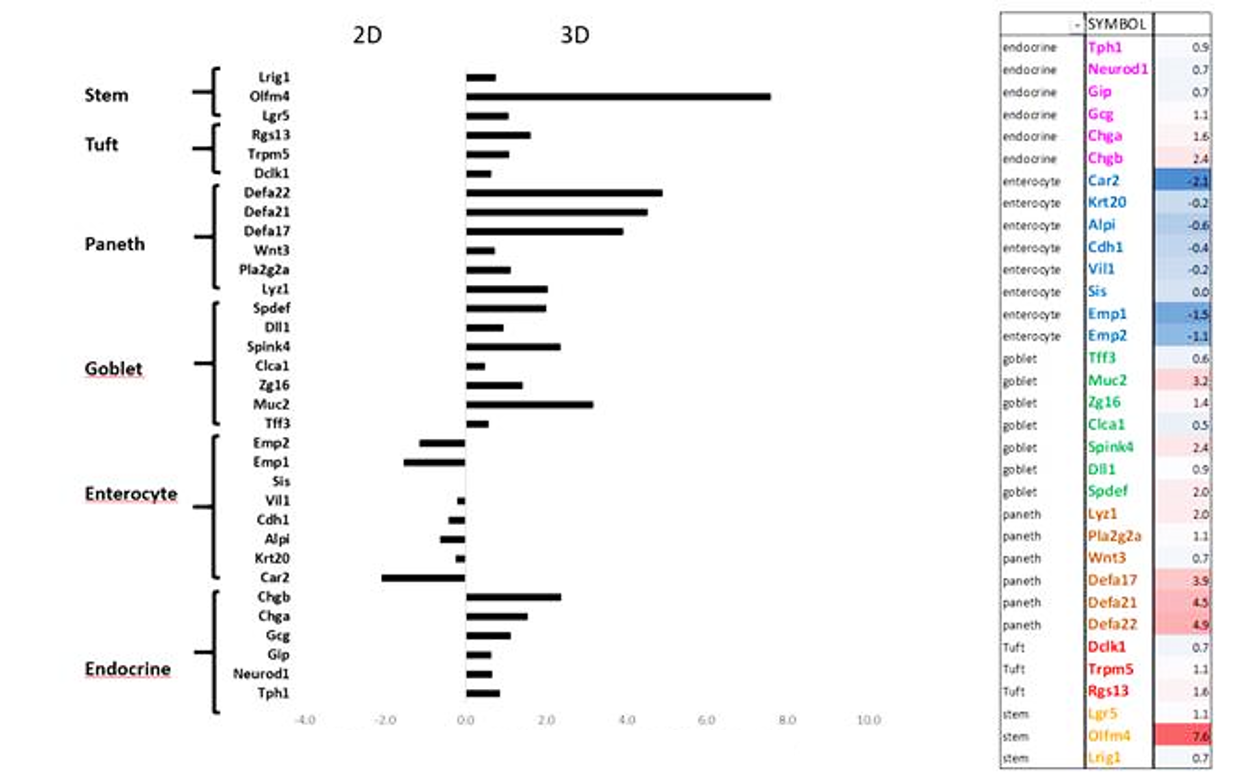


**Figure S1. Expression of cell-specific markers of 2D and 3D intestinal organoids.** Expression levels showed the presence stem cells, paneth, goblet, endocrine cells and to a lower extend also enterocytes in organoids cultured in a 3D fashion. Expression of Stem, Paneth, Goblet and endocrine cell-specific markers in 3D, which were reduced in 2D, and markers for enterocytes, although present in 3D, were increased when organoids were cultured in 2D.
