# Supplementary material for: Development of a self-limiting model of methotrexate-induced mucositis reinforces butyrate as a potential therapy
Source: Sci Rep. 2021 Nov 25;11:22911. doi: 10.1038/s41598-021-02308-w (PMC8617074; doi:10.1038/s41598-021-02308-w)
Supplement: Supplementary file 2 — Supplementary Information. [file 41598_2021_2308_MOESM2_ESM.docx]

**Supplementary Materials and Methods**

**Crypt isolation and 2D-organoid culture**

Crypt isolation was performed according to methods described before ^11,13^. A female mouse C57/BL6, 6 months of age was sacrificed using carbon dioxide. The intestine was removed and the ileum isolated. Villi were removed from the ileum by scraping with a scalpel and the tissue was cut into small pieces, transferred to a tube with ice-cold PBS and washed three times with PBS (spun down at 335x g for 5 minutes between every wash step). A Polter-Elvehjem tube was then used to fragment the tissue and the cell suspension was transferred to a tube with ice-cold PBS, washed three times with PBS and subsequently filtered through a 100 μm filter. The now isolated crypts were spun down at 335x g for 5 minutes at room temperature and supernatant was removed. The crypts were resuspended in Matrigel (Corning; 356231), and drops of 25 μl suspension were added to each well (Corning Costar). The plates were then incubated for 15 minutes at 37 ˚C until the droplets had solidified. One ml of organoid culture medium supplemented with 10% FCS, 10 μM Y27632 (Selleckchem; S1049), CHIR 4,3 μM, (Sigma; SML 1046) and 2-Propylpentanoic acid (Sigma; P6273; 1:8000). After 3 days of incubation at 37˚C and 5% CO2, medium was replaced by normal culture medium: DMEM/F12 medium containing GlutaMAX supplement, 1mM sodium pyruvate, MEM Non-Essential Amino Acids, 100U/ml Penicillin-Streptomycin, R-spondin 1 (Homebrew), WNT (Homebrew) and the BMP4 inhibitor DMH1 0.5ug/ml (Sigma; D8946) to stimulate organoid formation. These organoids were passaged once a week in a 1:4 ratio.

Secretion assays, stainings and QPCR analysis were performed by plating organoids as described above. 24 wells plates were coated with a thin layer of 3 μg/ml rat tail Collagen I (IBIDI GmbH; 50201). After solidification of the Collagen I coating, a suspension of organoids in medium supplemented with 10% FCS, 10 μM Y27632 (Selleckchem; S1049), CHIR 4,3 μM, (Sigma; SML 1046) and 2-Propylpentanoic acid (Sigma; P6273; 1:8000) was added to each well. The organoids were grown for 2 days at 37°C and 5% CO2.
